# Emerging toxicological awareness of per- and polyfluoroalkyl substances: the rising concern over ‘forever chemicals’

**DOI:** 10.1242/dmm.052647

**Published:** 2025-11-25

**Authors:** Jamie C. DeWitt, Gretta Goldenman, Rainer Lohmann, Carla A. Ng, Zhanyun Wang

**Affiliations:** ^1^ Department of Environmental and Molecular Toxicology, Oregon State University, Corvallis, OR 97331, USA; ^2^ Milieu Consulting SPRL (retired), Brussels 1060, Belgium; ^3^ Graduate school of Oceanography, University of Rhode Island, Kingston, RI 02881, USA; ^4^ Department of Civil and Environmental Engineering, University of Pittsburgh, Pittsburgh, PA 15261, USA; ^5^ Empa-Swiss Federal Laboratories for Materials Science and Technology, St. Gallen CH-9014, Switzerland

**Keywords:** Per- and polyfluoroalkyl substances (PFAS), Perfluorooctane sulfonic acid (PFOS), Perfluorooctanoic acid (PFOA), Toxicity, Emerging contaminants

## Abstract

Per- and polyfluoroalkyl substances (PFAS) are often called ‘forever chemicals’. This colloquialism reflects that many PFAS are recalcitrant to environmental and metabolic degradation, leading to long environmental and biological half-lives. This persistence, a concerning characteristic of these synthetic substances, is also a reason they are used in many products and processes. Most PFAS have physical−chemical properties that enable them to withstand extreme conditions and make them useful for a range of applications, including as surfactants or coatings that confer oil-, stain- and water-repellency. This combination of persistence and wide use has resulted in extensive environmental contamination and the presence of PFAS in living organisms, leading to use restrictions. Increasing evidence of health effects has also led to implementation of health protective guidelines. In the United States, federal regulations enacted in 2024 limit levels of six PFAS in drinking water; in the European Union, a proposed restriction would control use, import and production of the vast majority of PFAS. This Perspective article summarizes how knowledge of toxicological hazards and health-related costs of PFAS has progressed in recent years, leading to actions to restrict PFAS uses.

## Teaser

**Summary:** This article explains how per- and polyfluoroalkyl substances (PFAS) became widespread environmental contaminants and subjects of intense scrutiny, economic concern, public and ecosystem health concern, and regulatory oversight.

## Introduction

Emerging contaminants have been defined as “newly identified synthetic or naturally occurring chemicals or biological agents that are detected in the environment and are potentially hazardous or recently determined to be hazardous to humans and ecosystems” (Wang et al., 2024). Per- and polyfluoroalkyl substances (PFAS), a broad class of nearly 15,000 synthetic chemicals used in many consumer and household products since the 1950s, are contaminants of increasing concern due to the hazards they pose to human health and the environment. PFAS are particularly persistent in the environment and in living organisms because their physical−chemical structures prevent most of them from being degraded by environmental or biological processes. While sporadic reports of PFAS detection in human blood were published between the 1960s and 1980s (Guy et al., 1976; Travis, 2024), widespread detection of PFAS in wildlife brought closer scrutiny to this group of chemicals in the early 2000s. The latter period coincided with a decision by 3M, a major producer of various PFAS, to phase out certain PFAS chemistries (Lindstrom et al., 2011). By this time, it was also apparent to environmental and analytical chemists that several PFAS were already environmentally widespread and present in human blood samples worldwide (Lindstrom et al., 2011). Thus, in less than 50 years since industrial-scale production began, commercial uses of PFAS led to near global contamination, including their presence within the bodies of living organisms. One serious concern in the early 2000s, therefore, was whether PFAS exposure was toxic to living organisms. Of further concern was whether the risk of adverse health outcomes to living organisms would increase due to previous and ongoing PFAS exposure from their presence in the environment and in products. These concerns remain today.

**Fig. 1.**
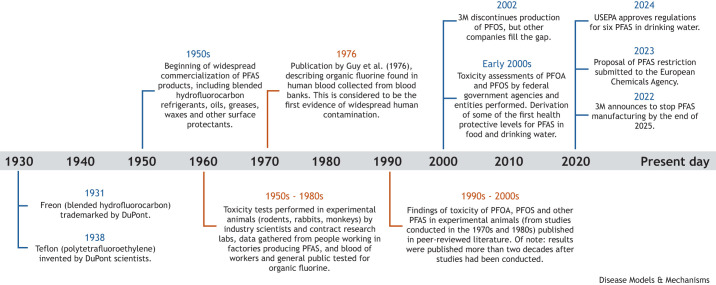
**Timeline of production, regulation and key toxicological findings regarding per- and polyfluoroalkyl substances (PFAS).** Blue lines represent production and regulatory activities; orange lines represent toxicological highlights. USEPA, United States Environmental Protection Agency.

The timeline of PFAS identification, synthesis and application into products and processes is illustrated in Fig. 1. PFAS were being synthesized in the 1930s and had been developed into various commercial uses by the 1950s. Accurate estimation of all current PFAS uses in products and processes is challenging for various reasons, including claims of confidential business information. However, a 2020 study identified more than 200 use categories and subcategories for more than 1400 individual PFAS (Glüge et al., 2020). Given what is now known about their hazards, justification for the continued use of PFAS in many products and processes is questionable, particularly in products such as cosmetics and ski waxes where their addition does not appear to be essential for the functionality of the product (Cousins et al., 2019). Notably, 3M announced that it would cease all PFAS manufacturing and work to discontinue use of PFAS across their product portfolio by the end of 2025 (see 3M News Center, 2022). However, if trends continue, this and other phase-outs in North America, Europe and Japan will continue to shift production of PFAS to emerging Asian economies (Wang et al., 2014).

## Emerging health concerns with delayed recognition

The timeline of emerging knowledge on toxicity of two commonly used PFAS, perfluorooctanoic acid (PFOA) and perfluorooctane sulfonic acid (PFOS), is foggy. PFOA and PFOS have been used in many different products and processes but notable uses of PFOA have included the production of fluoropolymers, such as Teflon™. PFOS was included as a major ingredient of Scotchgard^TM^ surface protectant and aqueous film-forming foams. The United States Environmental Protection Agency (USEPA) wrote a draft risk assessment of the potential human health effects of PFOA in 2005, in response to unexpected discoveries regarding PFOS toxicology and bioaccumulation. However, 3M had already discontinued production of PFOS in 2002 based on evidence of persistence, bioaccumulation in humans, and potential health and environmental risks (Lindstrom et al., 2011). Prior to 2000, few publications were available on toxicological properties of PFOA, PFOS or other PFAS, but internal company documents now publicly available revealed that adverse health outcomes had been observed in a range of experimental animals, including mice, rats, rabbits and monkeys (Grandjean, 2018). Some of these data are now in scientific publications or are part of public dockets maintained by the USEPA for PFOA and related PFAS, for example, despite much delayed disclosure to the broader scientific community and the public (Grandjean, 2018) (see also USEPA Risk Management for Per- and Polyfluoroalkyl Substances (PFAS) under TSCA). In one such toxicological study conducted in 1965 and sponsored by DuPont (a previous manufacturer of PFAS that spun off its fluoroproducts into the company Chemours in 2015), male and female albino rats were fed an unspecified PFAS at various doses for three months, although details of the specific PFAS was redacted from the report. Pathological changes in the liver, including increased liver weight and cell size, were observed in two dose groups (see USEPA Risk Management for Per- and Polyfluoroalkyl Substances (PFAS) under TSCA). The same USEPA guidance document contains various other sources of information on PFAS, including a 1979 internal memo from the then medical superintendent of a DuPont manufacturing facility in West Virginia, who noted workers exposed to PFOA “may possibly have positive liver function tests more often than the plant population as a whole” and suggesting a liver function survey should be undertaken. Thus, early evidence of PFAS health impacts existed but was not broadly accessible (Grandjean, 2018).

Perhaps one of the most well-known accounts leading to public knowledge of health and environmental effects of PFAS occurred in the legal arena and began in the late 1990s. Spearheaded by attorney Rob Bilott from the Taft law firm in Cincinnati, Ohio, USA, a class action lawsuit against DuPont eventually afforded a community impacted by PFAS contamination the opportunity to become part of health studies and to receive medical monitoring (see Taft law firm – case studies 2023). The lawsuit also facilitated the uncovering of records of early industry studies and triggered further health research. The C8 Health Project, funded by the class action settlement, gathered health-related information and blood samples from a pool of nearly 70,000 community members covered by the settlement (see C8 Science Panel). The C8 Health Project data identified several probable links between PFOA exposure and health risks – including kidney and testicular cancer, ulcerative colitis, thyroid disease, high cholesterol and pregnancy-induced hypertension – that, even today, form foundational knowledge of adverse health effects from exposure to PFAS, especially PFOA. For example, the Agency for Toxic Substances and Disease Registry (ATSDR) uses these probable links to highlight how PFAS exposure affects health (see ATSDR – How PFAS Impacts Your Health). Moreover, the C8 Health Project findings also provided a strong basis for clinical care recommendations made by a committee of the National Academy for Sciences, Engineering, and Medicine (National Academies of Sciences, Engineering, and Medicine et al., 2022).

**Fig. 2.**
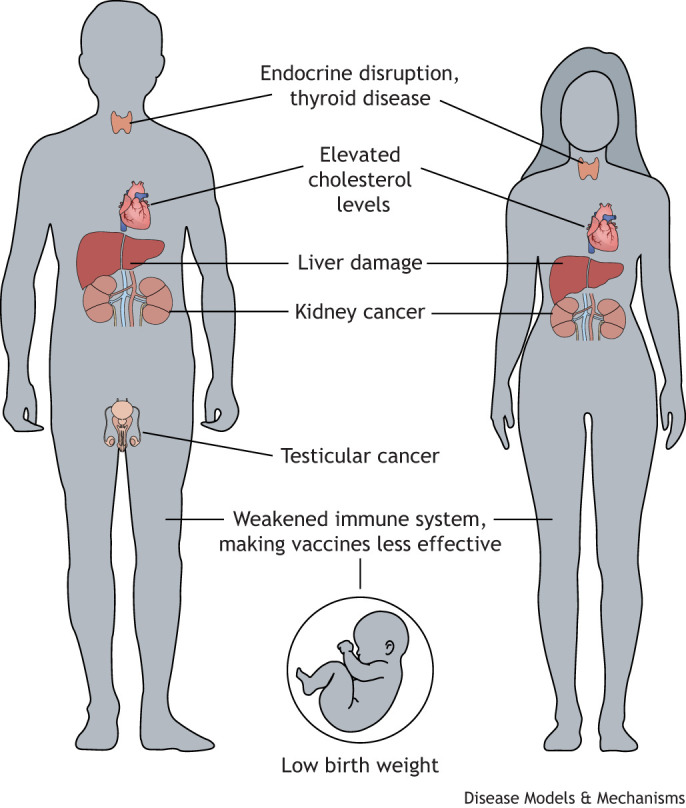
**Adverse health outcomes of exposure to per- and polyfluoroalkyl substances (PFAS) in humans.** Adverse health outcomes have been uncovered by toxicological studies in experimental animal models and epidemiological studies of exposed people. Figure adapted by DMM from original version created by the Pacific Northwest Center for Translational Environmental Health Research.

Additional studies by epidemiologists and toxicologists working in the PFAS space have identified a broad range of adverse health effects in exposed experimental organisms and humans, highlighted in Fig. 2. Many of the health risks uncovered across human populations have been confirmed in typical inbred rodent models that were given individual PFAS in food or water at specific doses and for defined durations. Although these adverse health effects are diverse, work in *in-vitro* models and in traditional rodent models, as well as in transgenic mice with modifications to a receptor known as the peroxisome proliferator activated receptor alpha (PPARA) have uncovered some potential mechanisms of PFAS toxicity (Fenton et al., 2021). It is now known that PFAS interact with cell membranes, mitochondria and molecular signals that control cell functions (Fenton et al., 2021). As an example of how mechanistic data underscore carcinogenic health risks of PFAS exposure, the International Agency for Research on Cancer (IARC) classified PFOA as a known human carcinogen and PFOS as a possible human carcinogen, citing immunosuppression and epigenetic modifications as mechanistic evidence supporting these classifications (IARC, 2025).

## Actions to protect public health

The community of science around PFAS has grown enormously over the past few decades, with the number of publications exponentially increasing in the last ten years (reviewed by DeWitt et al., 2024). Improved understanding of PFAS environmental fate and transport, and levels of PFAS in different environmental media have demonstrated that PFAS are nearly everywhere scientists look (Ackerman Grunfeld et al., 2024). Newly developed and sensitive analytical chemistry techniques have revealed overlooked and novel PFAS in drinking water supplies, indicating that living organisms are exposed to many more PFAS than just PFOA and PFOS (Hopkins et al., 2018; Kotlarz et al., 2020). The presence of both well-studied and understudied PFAS in the environment and in living organisms has led to great public concern about the health risks and costs to society posed by the harmful impacts of PFAS on human and ecosystem health (Obsekov et al., 2023). In response, some jurisdictions are implementing phase-outs, restrictions and health-protective regulations. In spring of 2024, the USEPA issued maximum contaminant levels (MCLs) and maximum contaminant level goals (MCLGs) for PFOA, PFOS and four other PFAS in drinking water under the Safe Drinking Water Act (SDWA) (see USEPA, SDWA). Under this rule, the MCLG for PFOA and PFOS in public drinking water is set at 0 ppt, reflecting their classification by the USEPA as ‘likely human carcinogens’ and indicating that no level is considered safe. However, due to technological and economic constraints, the enforceable MCL is set at 4 ppt, which corresponds to the current detection limit for these substances.

In their documents to support MCLs and MCLGs chosen for PFOA and PFOS, the USEPA also evaluated non-cancer health effects, which – although not the basis for the regulations – highlight the multi-system toxicity of PFAS. For PFOA, these non-cancer health effects included a weakened immune system, and for both PFOA and PFOS they included impacts on developing organisms and cardiovascular disease risk from elevated cholesterol (Fig. 2) (see USEPA Toxicity Assessment of PFOA, 2024 and USEPA Toxicity Assessment of PFOS, 2024). These health effects were reported in studies of exposed humans with support from numerous studies of experimental animals, highlighting the strength of the data on adverse health effects of PFAS exposure. These documents also note that additional studies to uncover the molecular mechanisms underlying identified health effects are needed to better understand how PFAS exposures affect living organisms. Very few studies have combined PFAS exposures with specific disease models; however, such approaches would provide insights into how PFAS exposures may blunt or exacerbate disease severity within specific mechanistic pathways represented by the disease models.

Additional actions to protect public health have been taken in the European Union (EU), Canada, individual states within the USA and in many other parts of the world. The state of Maine in the USA, for example, was the first state to pass a ‘PFAS in Products Program’ law that prohibits sales of products that contain intentionally added PFAS (Maine Department of Environmental Protection, see PFAS in Products). Several other states have followed suit in some form or another, restricting PFAS in various consumer products (see Safer States). In the EU, a proposal under consideration could result in a full PFAS ban or a ban with time-limited exemptions for limited PFAS applications (European Chemicals Agency news, 2025). It is important to mention that members of PFAS-exposed communities, and investigative journalists across the US and within the EU have been at the forefront of PFAS advocacy, educating decision-makers about scientific data, and demonstrating the extent of PFAS contamination in the environment and the potential impacts on human health.

## Conclusions

In under a century, PFAS have exceeded what some consider a planetary boundary for chemicals, such that there is no more “safe operating space for humanity” (Rockström et al., 2009; Cousins et al., 2022). Because levels of several PFAS in rainwater exceed drinking water guidelines in some countries, there is a real danger of adverse health effects to populations across the globe (Cousins et al., 2022). The phase-out of PFAS from all products by a major PFAS producer, 3M, demonstrates that elimination of PFAS from the supply chain can be a feasible, albeit challenging, goal. However, other PFAS producers continue to produce and market PFAS, which will inevitably result in ongoing exposures and environmental contamination. A key solution proposed by experts in the field is to rapidly minimize PFAS uses and releases wherever possible, so that society does not have to bear the rapidly increasing burden of exposure to these persistent, toxic chemicals (Cousins et al., 2022). In addition, environmental health scientists must continue to evaluate exposed human populations to uncover or describe additional health risks, investigate the molecular mechanisms of PFAS toxicity in multiple models, and find ways to prioritize toxicologically understudied types of PFAS for more in-depth analyses. These are essential to ensure that the best available science supports decision making on future management of PFAS.
